# Evaluating patient outcomes in South Australia's new pediatric eating disorder treatment program

**DOI:** 10.1186/2050-2974-2-S1-O13

**Published:** 2014-11-24

**Authors:** Mandy Yiu, Michael Batterham, Eva Vall

**Affiliations:** 1 Flinders Medical Centre, Adelaide, Australia; 2 Flinders University, Adelaide, Australia

## 

Eating disorder (ED) admissions to the Paediatric Inpatient Unit at Flinders Medical Centre increased sharply between 2008 and 2012. In response, the Paediatric Eating Disorder Program was established in February 2013. The multi-disciplinary program is based on the principles of the Maudsley approach, and consists of a 4-level system including medical stabilization, establishing normal eating and transition to home. To evaluate patient outcomes in the new program, a research project commenced in August 2013. In the six months to February 2014, 25 eligible patients were assessed. This patient group displayed severe ED psychopathology at a level consistent with clinical ED populations and significantly higher than matched non-clinical norms. Mean BMI at admission was 16.38 (SD = 2.05). After an average length of stay of 20.15 days (SD = 12.78), the mean discharge BMI was 18.19 (SD = 1.76). The number of readmissions has decreased since the program started: 39% of patients had more than one admission in 2013 compared to 47% in 2012. We present a sample case to highlight the complexities of this patient group, and to provide a practical description of the treatment approaches employed in the program.

This abstract was presented in the **Service Initiatives: Child and Adolescent Refeeding and FBT** stream of the 2014 ANZAED Conference.

